# *Entamoeba histolytica*: Gene Expression Analysis of Cells Invading Tissues

**DOI:** 10.1155/2014/364264

**Published:** 2014-01-27

**Authors:** Helen C. Fernandes, Ana F. Costa, Michelle A. R. Freitas, Almir S. Martins, Jorge L. Pesquero, Élida M. Rabelo, Maria A. Gomes

**Affiliations:** ^1^Department of Parasitology, Institute of Biological Sciences, Universidade Federal de Minas Gerais, 31270-901 Belo Horizonte, MG, Brazil; ^2^Laboratory of Parasitology, Institute of Biomedical Sciences, Universidade Federal de Uberlândia, 38400-902 Uberlândia, MG, Brazil; ^3^Department of Physiology and Biophysics, Institute of Biological Sciences, Universidade Federal de Minas Gerais, 31270-901 Belo Horizonte, MG, Brazil

## Abstract

*Entamoeba histolytica* is a protozoan parasite that presents a risk to the health of millions of people worldwide. Due to the existence of different clinical forms caused by the parasite and also different virulence levels presented by one strain, one would expect differences in the profile of gene transcripts between virulent and nonvirulent cultures. In this study we used the differential display to select gene segments related to invasiveness of amoeba. One Brazilian strain of *E. histolytica* in two conditions, able or not to cause lesions in experimental animals, was used. RNA from this strain, was used to study the differential expression of genes. 29 specific gene fragments differentially expressed in the virulent strain were selected. By real-time PCR, six of these genes had confirmed their differential expression in the virulent culture. These genes may have important roles in triggering invasive amoebiasis and may be related to adaptation of trophozoites to difficulties encountered during colonization of the intestinal epithelium and liver tissue. Future studies with these genes may elucidate its actual role in tissue invasion by *E. histolytica* generating new pathways for diagnosis and treatment of amoebiasis.

## 1. Introduction

*Entamoeba histolytica,* the protozoan responsible for amoebiasis, a disease that affects millions of people worldwide [1, 2], presents great diversity of clinical manifestations ranging from asymptomatic intestinal infections to intestinal and extra-intestinal invasion. It is speculated that the result of the infection constitutes a multifactorial event mainly determined by two factors: the potential pathogenic of *E. histolytica* strain and the host immune response [3]. Among factors related to the parasite, the profile of gene transcription has been extensively studied. Biochemical and molecular differences between virulent and nonvirulent strains have been described [4]. Recent research pointed to increased gene expression of molecules related to tissue lysis, phagocytosis, and motility in invasive amoebas. Among these are pore-forming proteins, phospholipase A, and cysteine proteinases [5–7]. Differences in the virulence of strains maintained in different culture conditions [8], like passage through liver hamster [9] and prolonged axenic culture [10], were also reported. These data suggest a modulation of gene expression during development of invasive amoebiasis and that this process is regulated by multiple and complex pathways. It is believed that not all genes involved in the invasive process are known. Therefore, the analysis of gene expression in different strains of *E. histolytica* and in different culture conditions is extremely important for a better understanding of the biology of this parasite since give us data to support the participation of new and already known factors on its virulence. In this context, the purpose of this study was to identify genes differentially expressed in trophozoites of the same strain of *E. histolytica* under different virulence conditions.

## 2. Materials and Methods

### 2.1. Strain of *Entamoeba histolytica*

The strain ICB-CSP (CSP), previously identified as *E. histolytica* [10], was chosen because it was isolated from a patient with dysenteric colitis and also to present high capacity to cause lesions in tissues in experimental models. During maintenance on axenic culture, this strain had its virulence attenuated, losing their ability to cause lesions in tissues.

### 2.2. Virulence Activation

To activate the virulence, the trophozoites (1 × 10^6^) were inoculated in the left lobe of the liver of hamsters (*Mesocricetus auratus*). After 24 hours, the animals were sacrificed and liver fragments were grown in culture medium for reisolation of trophozoites. This strain was renamed as reisolated CSP (CSP-R).

### 2.3. Reverse Transcription

Total RNA was extracted (Trizol Reagent, Life Technologies, USA) from trophozoites (1 × 10^6^ cells of strains CSP in culture and CSP-R), isolated directly from hamsters liver. The RNA was resuspended in 0.01% DEPC water, quantified by spectrophotometry, and stored in a freezer at −80°C until use. Three microliters of RNA treated with deoxyribonuclease I (Life Technologies, USA) was used for cDNA synthesis. The reaction contained 25 ng/*μ*L oligo (dT)_12–18_, 500 *μ*M each dNTP, 75 mM KCl, 3 mM MgCl_2_, 10 mM DTT, and 200 units of reverse transcriptase (SuperScript II RNase H-reverse transcriptase, Invitrogen, USA) in 50 mM Tris-HCl buffer (pH 8.3) in a final volume of 10 *μ*L. The reaction was carried out at 42°C for 50 minutes, as negative control samples were also prepared without addition of reverse transcriptase. Differences in expression levels between CSP and CSP-R were analyzed using the RNA differential display as previously described [11]. For PCR a single random primer was used (Table 1).

### 2.4. Polymerase Chain Reaction

PCR was carried out in 10 mM Tris-HCl pH 8.4 buffer containing 50 mM KCl, 1.5 mM MgCl_2_, 0.1% Triton X-100, 200 *μ*M each dNTP, 2.6 *μ*M of random primers (Table 1), and 0.12 units of Taq DNA polymerase (Phoneutria, MG, Brazil) plus 1 *μ*L of cDNA sample in a final volume of 10 *μ*L. Samples were submitted to two cycles of 2 minutes at 95°C, 1 minute at 37°C, and 2 minutes at 72°C, followed by 29 cycles of 1 minute at 95°C, 1 minute at specific temperature for each primer, and 2 minutes at 72°C. The reaction product was loaded on nondenaturing 6% polyacrylamide gel (16 mm × 13 mm × 1 mm), in TAE buffer (Tris-acetate-EDTA), under an electric field of 100 volts. The gels were stained with silver nitrate [12]. Fragments of gel containing the bands with the differentially expressed genes were transferred to microfuge tubes in which were added 10 mM Tris-HCl pH 8.4 buffer containing 50 mM KCl, 1.5 mM MgCl_2_, 0.1% Triton X-100. This mixture was heated at 95°C for 20 minutes. The cDNAs extracted from the gel were used in a reamplification PCR using 10 mM Tris-HCl pH 8.4 buffer containing 50 mM KCl, 1.5 mM MgCl_2_, 0.1% Triton X-100, 200 *μ*M each dNTP, 0.6 *μ*M of one primer, and 1.0 unit of Taq DNA polymerase (Phoneutria, MG, Brazil). The program used was similar to that described above excluding the initial cycle of annealing at 72°C. The products were loaded on a 1% agarose gel stained with ethidium bromide (0.5 *μ*g/mL) and purified from the gel using the GFX kit (Amersham Pharmacia Biotech Inc.) according to manufacturer's instructions. The purified cDNA was cloned into pGEM-T easy vector (Promega, Madison, WI, USA) and transformed into *Escherichia coli*, strain DH5*α*. Positive clones were selected using the system ampicillin/IPTG/X-Gal [13]. The size of cloned fragments was confirmed by PCR.

### 2.5. DNA Sequencing and Sequence Analysis

Recombinant plasmids were isolated using Wizard Plus SV kit minipreps (Promega, Madison, WI, USA) and submitted to sequencing reaction. The sequencing reaction was performed according to the method previously described [14]. Reaction products were subjected to the sequencer ABI PRISM 3130. Each clone was sequenced two times in both directions and the sequences were compared with those deposited at the National Center for Biotechnology Information (http://www.ncbi.nlm.nih.gov/BLAST) and Sanger Institute (http://www.sanger.ac.uk/cgi-bin/blast/submitblast/e_histolytica) using the Basic Local Alignment Search Tool program [15].

### 2.6. Real-Time PCR

The Kit SYBR Green PCR Master Mix (Power SYBR Green PCR Master Mix, Applied Biosystems, Foster City, CA, USA) was used. The primers for all sequences were designed using Primer Express V2.0 program (Applied Biosystems, USA) to generate fragments between 70 and 190 bp. All primers were previously tested in conventional PCR using the plasmids containing the cloned fragment as a positive control. As internal control, the expression levels of the actin gene was determined [16]. The reactions were performed in 96-well plates, in a final volume of 25 *μ*L containing Power SYBR Green PCR Master Mix (Applied Biosystems, USA), 0.45 *μ*M each primer, and 1 *μ*L of cDNA. Amplification conditions consisted of 40 cycles of 95°C/15 s, 60°C/45 s, and 72°C/30 s. Reactions were carried out in duplicate. The dissociation stage was added to the program for which the analysis of specific amplification could be made after completion of reactions. For relative quantification of amplification products the method 2^−ΔΔCt^ was used [17].

## 3. Results

**Figure 1:**
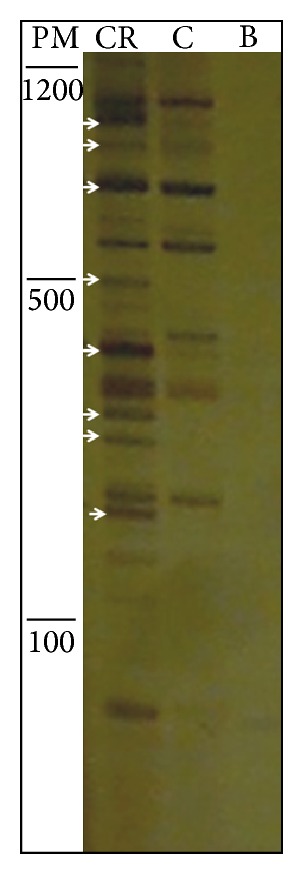
Differential display. Electrophoretic profile of differential display between virulent (CR) and nonvirulent (C) cultures of the strain CSP obtained in 6% polyacrylamide gel. B is PCR negative control and PM is molecular mass standard in base pairs. The arrows show cDNA fragments of higher intensity in the virulent strain.

**Figure 2:**
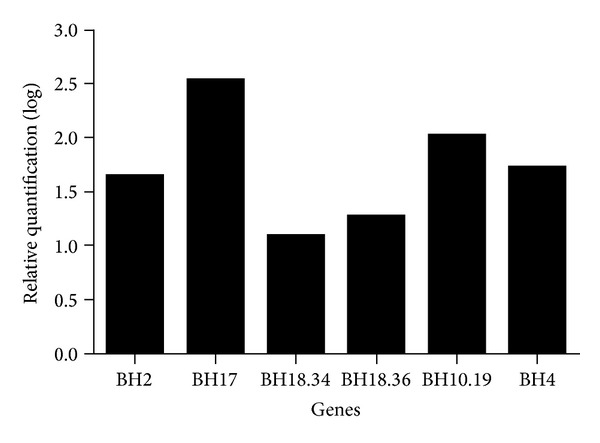
Comparison of quantitative RT-PCR data (log_10_). Relative quantification of transcription in virulent versus nonvirulent *E. histolytica *cultures.

The CSP strain had its virulence activated after the second passage through hamster liver. The lesions produced belong to the class IV as previously defined [18]. Only two of the primers used (Kcal 2 and RB1.1) showed no differences in the amplification profiles between CSP and CSP-R.  Figure 1 illustrates an example of the differential profile using ERR1 primer. 29 fragments with higher levels of expression specific to virulent culture, the CSP-R, with sizes ranging from 170 to 1150 bp (BH1 to BH29) were identified. These were extracted from the polyacrylamide gel and used in a reamplification reaction with the same primers they generated. Eighteen fragments do not generate any product or produce more than one fragment, and therefore were discarded. Eleven fragments showed products of the expected size and were successfully cloned. At least three colonies of each fragment were subjected to PCR to confirm the size of the inserts. Those which showed the expected size were submitted to sequencing. For some plasmids more than one sequence was obtained indicating comigration of the fragments. From the original 11 fragments 13 different sequences were obtained. One sequence showed no similarity with any of the genes present in the database and therefore was excluded from the study. Primers were designed for the 12 sequences that showed similarity with any gene present in the NCBI database (Table 2). All 12 pairs of primers were tested in a conventional PCR using the plasmids containing cloned fragments, used as positive controls, and cDNA products obtained from cultures in different conditions. All fragments were amplified (data not shown). However, in the real-time PCR only, six fragments (BH2, BH4, BH10.19, BH17, BH18.34, and BH18.36) were successfully amplified. The corresponding DNA sequences were analyzed for peptide sequence prediction by SignalP 3.0 Server program available on http://www.cbs.dtu.dk/services/. Only the sequence BH10.9 showed signal peptide prediction. The genes showed differential expression in the range of 12.1- to 339-fold more expressed in the virulent culture (CSP-R) compared to the nonvirulent culture (CSP) (Figure 2).

## 4. Discussion

It is well established that *E. histolytica* is a pathogenic organism in which its virulence varies according to environmental conditions [19]. Therefore, studies of the transcription profile and changes in gene expression under different conditions are important for understanding the pathogenesis of these parasites and physiology including regulation of the life cycle stages of differentiation, development, and tissue invasion. In this context, the identification and characterization of differential gene expression may reveal important molecular markers in the events mentioned above. Different techniques have been used in studies to determine gene expression differences, such as differential display, subtractive hybridization of cDNA libraries, SAGE (serial analysis of gene expression), and cDNA microarrays [20–23].

Alterations in the expression pattern of molecules related to virulence may help to define invasiveness markers. Previous research has demonstrated differences in gene expression in trophozoites of *E. histolytica* presenting different virulence conditions [4–10, 16, 24–26]. However, they compared the pathogenic isolate HM-1:IMSS with the nonpathogenic Rahman or different cell lines of HM-1:IMSS. In this study we compared the gene expression of a Brazilian isolate of *E. histolytica* with high aggressiveness to experimental animals. This strain had its virulence attenuated by prolonged cultivation becoming unable to injury tissues. Its virulence was activated by inoculation into hamster liver. As the attenuated and activated isolates were taken from axenic culture, the differences found in the strain invading tissues can reveal new molecules involved in amoebic pathogenicity, in addition to confirmation of virulence genes found by other authors. For gene expression evaluation, the RNA differential display technique was used to be simple and allow the analyses of a large number of different transcripts from a small amount of RNA [20]. 29 segments that were more expressed in the virulent culture of *E. histolytica* (CSP-R) was selected. From 13 sequences that were successfully reamplified, 12 had their level of expression confirmed by semiquantitative RT-PCR. However, only 6 had their expression level successfully analyzed by RT-qPCR.

SYBR green, a nonspecific fluorescence marker that binds to double-stranded DNA, was used in RT-qPCR. The SYBR contains in its structure a quaternary nitrogen positively charged, responsible for binding to DNA. We speculate that either the sequence composition or the amount and chemical characteristics of ions present in the samples could have interfered with the SYBR binding to the DNA. As a result, the fluorescence intensity would decrease, making detection of low expressed genes impractical. This may have occurred with the nonamplified fragments in RT-qPCR in this study.

One of the more expressed sequences of virulent culture is that of coding for acid phosphatase gene (fragment BH4). The acid phosphatase has been described in parasites such as *Leishmania mexicana* and *Trypanosoma*, being an essential enzyme for recycling membrane and endocytosis [4, 19], and may have a similar function in *E. histolytica*. Another gene more expressed in CSP-R is the Cdc48-like protein (fragment BH18.36). This gene is also more expressed in *E. histolytica* stimulated by type I collagen and calcium ATPase activity presenting and participating in the processes of endo- and exocytosis [27]. *E. histolytica* is known for its high content of vesicles containing aggressive molecules such as lysozyme, pore-forming peptides, and proteinases.

Taking into account that it was evaluate transcripts of the same strain, capable (CSP-R) and unable (CSP) to cause lesions, the increased expression levels of genes encoding proteins such as acid phosphatase and EhCdc48-like, which are important elements in the regulatory mechanism of endocytosis, fusion of lysosomes, and recycling of membrane, suggest that these proteins may have important role in tissue invasion by the amoebae.

Four distinct sequences encoding hypothetical proteins (fragments BH2, BH10.19, BH17, and BH18.34) had also confirmed the differential expression, all of them more expressed in the virulent culture. One of these proteins had signal peptide, should be secreted, and may have a more direct action in the process of tissue damage in the host.

## 5. Conclusions

Differences in gene expression presented here as well as those described by others may be related to adaptation of trophozoites due to difficulties during the colonization of the intestinal epithelium and liver tissue. Future studies of these genes may elucidate its actual role in amoebic tissue invasion, generating new pathways for diagnosis and treatment of amoebiasis.

## Figures and Tables

**Table 1:** Oligonucleotides used in the differential display reaction.

Oligo	Sequence
AP1	5′ACAGGATTAATTAATACATTAGAAAAT3′
AP2	5′ACAGGACTTATTAATACACCTTGAAAAT3′
AP3	5′ACTGGTTTAATTAATACTTTAGAAAAT3′
ERR1	5′CGCTCAAAATATCCACTTCTAC3′
RD5	5′ATCTGGTTGATCCTCCTGCCAGT3′
Kcal 1	5′GCGGCCGCTCAGGGGTTTTCCTTC3′
Kcal 2	5′CTCGAGAAAAGAGTTGTTGGAGGATATAAC3′
GR	5′CTCGAGAAAAGAGTTGTTGGAGGATATAAC3′
Edmt1	5′TATTATAATGGCTTTATTTTG3′
PF	5′TTCAACTCTGTGAGATGAATGC3′
CARBOXI-U	5′CAGAGTGACCCTGCCTGC3′
RB1.1	5′CAGGTGTGTGAGCATGGGC3′

**Table 2:** Similarities of fragments more expressed on the virulent strain obtained in the blast.

Fragment	Size/homology	Protein and access number	Identity	Score
BH2	251 bp/1–249	Hypothetical protein of *Entamoeba histolytica* HM-1:IMSS XP_655762.1	100%	166
BH4	474 bp/104–304	Acid phosphatase of *Entamoeba histolytica* HM-1:IMSS XM_644626.1	96%	283
BH6.2	182 bp/47–113	Hypothetical protein of *Entamoeba histolytica* HM-1:IMSS XM_643628.1	90%	87.9
BH6.4	187 bp/3–134	Hypothetical protein of *Entamoeba histolytica* HM-1:IMSS XM_652098.1	94%	198
BH7	377 bp/3–374	Calcium-gated potassium channel protein of *Entamoeba histolytica* HM-1:IMSS XP_655083.1	97%	203
BH10.19	833 bp/1–617	Hypothetical protein of *Entamoeba histolytica* HM-1:IMSS XM_645643.1	98%	402
BH10.22	414 bp/162–413	Ras guanine nucleotide exchange factor of *Entamoeba histolytica* HM1:IMSS XP_649781.1	95%	177
BH11	180 bp/1–96	Plasma membrane calcium-transporting ATPase of *Entamoeba histolytica* HM-:IMSS XP_651287.2	100%	68.2
BH17	223 bp/2–199	Hypothetical protein of *Entamoeba histolytica* HM-1:IMSS XP_656432.1	100%	117
BH18.34	255 bp/3–254	Hypothetical protein of *Entamoeba histolytica* HM-1:IMSS XP_001914291.1	94%	409
BH18.36	102 bp/3–101	Cdc48 similar protein of *Entamoeba histolytica* gb AAF74998.1	64%	25.8
BH23	115 bp/1–115	Small-conductance mechanosensitive ion channel of *Entamoeba histolytica* HM-1:IMSS XM_650592.1	100%	213
